# High sustained virologic response rates of sofosbuvir-based regimens in Chinese patients with HCV genotype 3a infection in a real-world setting

**DOI:** 10.1186/s12985-019-1184-y

**Published:** 2019-06-03

**Authors:** Qunying Han, Xiude Fan, Xiaoyun Wang, Ye Wang, Huan Deng, Xiaoge Zhang, Kun Zhang, Na Li, Zhengwen Liu

**Affiliations:** Department of Infectious Diseases, First Affiliated Hospital of Xi’an Jiaotong University, No. 277 Yanta West Road, Xi’an, 710061 Shaanxi Province People’s Republic of China

**Keywords:** Hepatitis C, Genotype 3, Cirrhosis, Renal impairment, Treatment

## Abstract

**Background:**

Patients with hepatitis C virus (HCV) genotype 3 infection remain a difficult-to-cure population. This study evaluated the efficacy and safety of sofosbuvir-based regimen in genotype 3 patients in a real-world setting.

**Methods:**

HCV genotype 3a-infected adults with compensated liver disease were treated with sofosbuvir (SOF)/velpatasvir (VEL) or SOF/daclatasvir (DCV) with or without ribavirin (RBV) for 12 or 24 weeks, respectively. Efficacy was measured by sustained virologic response at post-treatment week 12 (SVR12). Adverse events were evaluated throughout the treatment and follow-up course.

**Results:**

A total of 41 genotype 3a-infected patients were included. Of them, 10 patients (24%) had cirrhosis, 3 (7%) had renal impairment, and 2 (5%) failed previous treatment. Nine patients (22%) were treated with SOF/VEL and 32 (78%) with SOF/DCV with or without RBV. SVR 12 was achieved in 100% (9/9) of patients treated with SOF/VEL for 12 weeks and in 97% (31/32) of those treated with SOF/DCV for 12 or 24 weeks. RBV addition and extension of treatment duration did not improve the SVR of SOF/DCV (RR: 1.04; *P* = 0.99 and RR: 1.09; *P* = 0.375, respectively). Ten patients with cirrhosis, 1 on hemodialysis and 2 with treatment-experience achieved SVR12. One treatment-naïve non-cirrhotic patient on hemodialysis treated with SOF/DCV for 24 weeks relapsed at week 8 post-treatment. No serious adverse events and relevant laboratory abnormalities were observed.

**Conclusion:**

SOF/VEL and SOF/DCV are highly efficacious and well tolerated in genotype 3a-infected patients with or without cirrhosis. RBV coadministration and extension of SOF/DCV treatment appear to add no improvement for efficacy.

**Electronic supplementary material:**

The online version of this article (10.1186/s12985-019-1184-y) contains supplementary material, which is available to authorized users.

## Background

Hepatitis C virus (HCV) infection is a major cause of liver cirrhosis, hepatocellular carcinoma (HCC), liver transplantation, and liver-related death worldwide [1]. It is estimated that the total global HCV prevalence is 2.5% and 177.5 million adults are infected by HCV [2]. The prevalence of HCV in China is not precisely known owing to the absence of large population-based studies. A national survey conducted in 1992 showed that the HCV infection rate was 3.20% in general population in China [3]. A review published in 2015 showed that the HCV prevalence in the general population was 1.6% in mainland China and 1.8–5.5% in Taiwan [4].

There are seven major HCV genotypes (HCV 1–7) including numerous subtypes [5]. Genotype 1 is the most prevalent worldwide (49.1%), followed by genotypes 3 (17.9%), 4 (16.8%) and 2 (11.0%) [2]. In China, six HCV genotypes have been found [6] and the major genotypes include genotypes 1, 2, 3, and 6 [7, 8]. The major subtypes are 1b, 2a, 3b, 6a and 3a [7, 8], accounting for 98.84% of the patient samples investigated (1b, 52.18%; 2a, 28.69%; 3b, 7.06%; 6a, 6.41% and 3a, 4.62%) [7]. Among the HCV genotypes worldwide, genotype 3 is the second most common genotype [2]. In China, genotype 3 is also among the major genotypes [7, 8] and is the most prevalent genotype in drug users [9]. Moreover, genotype 3 shows an increasingly wide geographic distribution over time [10] and is associated with a trend of earlier cirrhosis incidence [11].

HCV genotype is important in predicting the risk of disease progression and the response to treatment [12]. Genotype 3 is related to a higher rate of disease progression and mortality compared to other genotypes [13–15]. Genotype 3 is also associated with accelerated fatty liver disease [15], liver fibrosis [15, 16], cirrhosis [17] and a higher incidence of HCC [15, 17–19]. Moreover, genotype 3 has the lowest rate of sustained virologic response (SVR) when treated with the pegylated-interferon-based regimen [13]. Therefore, more effective treatments are needed for patients with genotype 3 infection.

With the advent of direct-acting antivirals (DAAs), the treatment of HCV infection has been greatly revolutionized. The current treatment recommendation for genotypes 1–6 infection (pangenotypic regimen) by the American Association for the Study of Liver Diseases (AASLD 2018) and the Infectious Diseases Society of America (IDSA) is a combination of glecaprevir (GLE)/pibrentasvir (PIB), sofosbuvir (SOF)/velpatasvir (VEL) or SOF/VEL/voxilaprevir (VOX) [20]. The European Association for the Study of the Liver (EASL 2018) recommends a combination of SOF/VEL or GLE/PIB [21]. The Asian-Pacific Association for the Study of the Liver (APASL 2016) recommends the use of SOF with ledipasvir (LDV) for treating all genotypes except genotype 3 [22]. For the treatment of HCV genotype 3 infection, AASLD (2018) and IDSA recommend a combination of SOF/daclatasvir (DCV), GLE/PIB, SOF + elbasvir/grazoprevir, SOF/VEL or SOF/VEL/VOX [20], EASL (2018) recommends SOF/VEL, GLE/PIB or SOF/VEL/VOX [21] and APASL (2016) recommends SOF/DCV [22]. Some modifications in the regimens may apply according to the disease severity, treatment-experience and comorbidities.

In China, the current use of DAAs for treatment of HCV infection varies according to the availability and price of the drugs and the willingness of the patient with full discussion on the justifiability by the patient and the consulting physician, in addition to the HCV genotypes infected and the disease condition, because some regimens of DAAs such as GLE/PIB and SOF/VEL/VOX have not been available and the medical expenses of DAAs have not been included in the reimbursement of health insurance in most areas.

Patients with HCV genotype 3 infection remains a difficult-to-cure population and HCV genotype 3-infected patients including those with cirrhosis, prior treatment failure or comorbidity may require different regimens. The present study aimed to evaluate the efficacy and safety of SOF-based regimens in patients with HCV genotype 3a infection in a real-world setting.

## Methods

### Study design and participants

This study included HCV genotype 3a-infected adults who had compensated liver disease and treated in the period from March 2016 to November 2017. Eligible participants were patients with chronic HCV genotype 3a infected compensated liver disease (with or without cirrhosis). Patients with renal impairment were also permitted. Patients who had one of the following criteria were excluded: age less than 18 years; co-infection with other genotype HCV; infection of hepatitis A, B or E or human immunodeficiency virus; autoimmune hepatitis, primary biliary cholangitis or primary sclerosing cholangitis, and decompensated liver disease including decompensated cirrhosis, liver failure and HCC. The patients were treated with sofosbuvir 400 mg plus velpatasvir 100 mg (SOF/VEL) daily for 12 weeks or sofosbuvir 400 mg plus daclatasvir 60 mg (SOF/DCV) daily with or without ribavirin (RBV) for 12 or 24 weeks, respectively, according to the availability of treatment option and the discretion of the patient and referring physician by discussion on the efficacy, safety consideration and cost. For patients on hemodialysis, the drugs of the day of hemodialysis were administered within 1 h before dialysis. All the patients consented to the treatments. The study was approved by the Ethics Committee of the First Affiliated Hospital of Xi’an Jiaotong University and was performed in a manner conforming to the Declaration of Helsinki. The requirement for written informed consent was waived because of the retrospective design of the study and the anonymous use of the data and elimination of the patients’ identification information.

### Assessments of efficacy and safety

The patients were carefully monitored for clinical symptoms and adverse events (AEs) including serious adverse events (SAEs) during the treatment and follow-up. Laboratory data including routine blood tests, biochemical liver and renal function and HCV RNA were regularly monitored at baseline, week 4 of therapy, the end of therapy (week 12 or week 24), and at week 12 after the completion of therapy. Additional laboratory tests might be performed in some patients with the request of patient and the consultation of referring physician. Efficacy was measured by sustained virologic response at post-treatment week 12 (SVR12). Serum HCV RNA was quantified by reverse-transcription polymerase chain reaction (RT-PCR) using the Cobas AmpliPrep/COBAS TaqMan HCV Test (Roche Diagnostics, Branchburg, NJ, USA), which has a lower limit of 15 IU/mL for HCV RNA quantification. HCV genotype was determined using RT-PCR with genotype-specific primers from the 5′noncoding region of the virus. Routine blood tests and biochemical liver and renal functions were determined using standard procedures. The primary endpoint was SVR12, which was defined as serum HCV RNA undetectable at 12 weeks after the end of therapy. The secondary endpoints were the treatment-related AEs and laboratory abnormalities.

### Statistical analysis

Categorical data were expressed as numbers (percentages) and continuous variables were expressed as mean ± standard deviation (SD). Serum HCV RNA levels are presented as log transformations. Continuous variables with normal distributions were analyzed using Student’s t test or the Mann-Whitney test. The paired t test was used to compare continuous variables. A *P* value < 0.05 was considered statistically significant. Statistical analysis was performed using SPSS 20.0 software (SPSS Inc., Chicago, IL, USA).

## Results

### Characteristics of the study population and treatment regimens

**Fig. 1:**
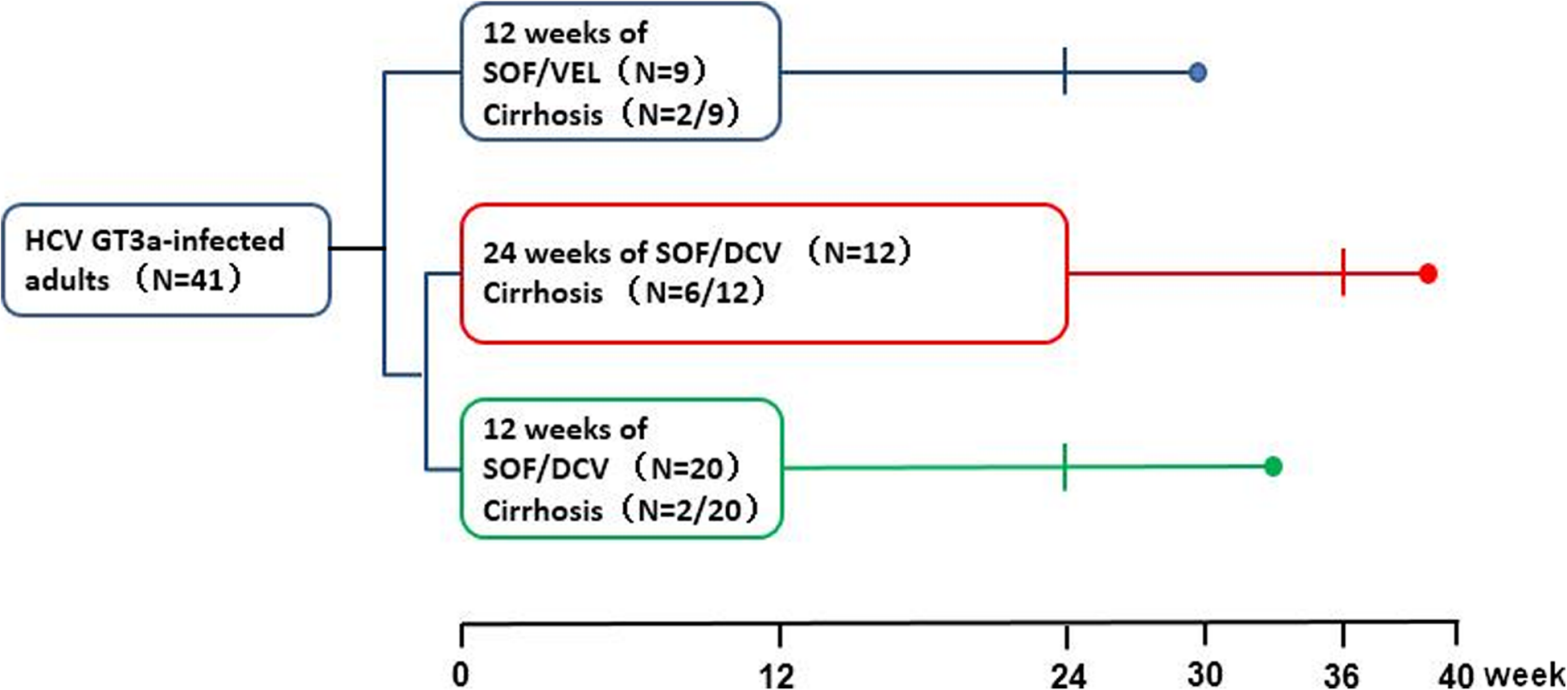
Study design for HCV genotype 3a infected patients enrolled in the study. Nine patients (including 2 with cirrhosis) were treated with SOF/VEL for 12 weeks, 20 patients (including 2 with cirrhosis) were treated with SOF/DCV for 12 weeks and 12 patients (including 6 with cirrhosis) were treated with SOF/DCV for 24 weeks. All the patients were followed-up for more than 12 weeks after the completion of treatment

**Fig. 2:**
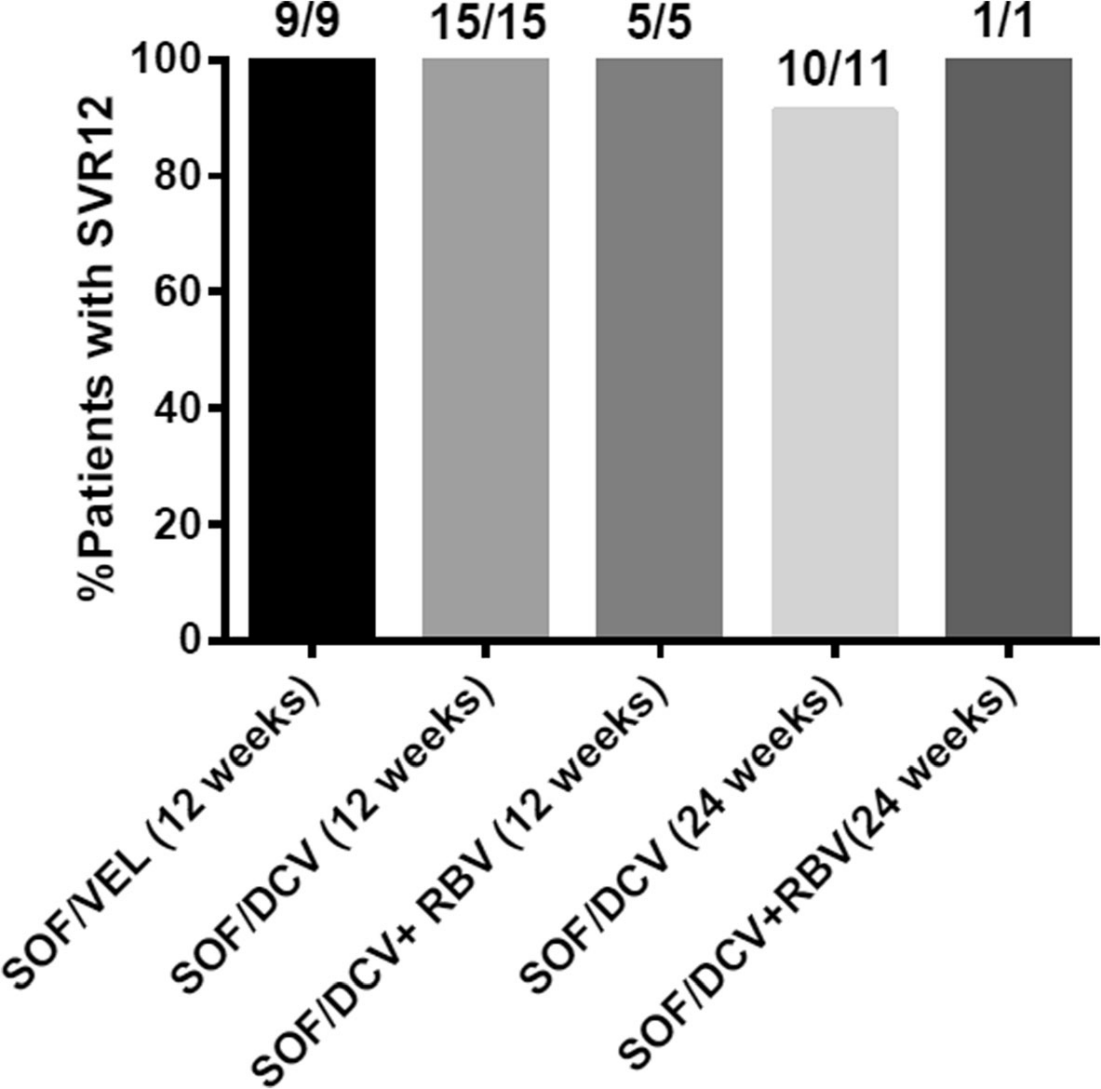
Percentage of patients who have achieved SVR12 with the different treatments analyzed

A total of 41 genotype 3a HCV infected patients were enrolled. Most of the patients were male (80%) and treatment-naïve (95%). Of the 41 patients, 26 (63%) had a history of intravenous drug abuse, 10 (24%) had cirrhosis, 3 (7%) had renal impairment (2 patients were on maintenance hemodialysis due to chronic renal failure and 1 had chronic glomerulonephritis and all of them were without cirrhosis), and 2 (5%) failed previous interferon/RBV treatment. Most of the patients chose SOF/DCV regimen because of the availability and lower price. Specifically, nine patients (22%) including 2 with cirrhosis were treated with SOF/VEL for 12 weeks. Thirty-two patients (78%) were treated with SOF/DCV. Of the 32 patients treated with SOF/DCV, 20 patients including 2 with cirrhosis were treated for 12 weeks and 12 patients including 6 with cirrhosis were treated for 24 weeks (Fig. 1, Table 1). Six (19%) patients treated with SOF/DCV in combination with RBV (5 were treated for 12 weeks and 1 for 24 weeks, Fig. 2).

**Table 1:** Baseline patient demographics and clinical characteristics

	Patients treated with SOF/VEL, 12 weeks (*n* = 9)	Patients treated with SOF/DCV (*n* = 32)		*P* value
		24 weeks (*n* = 12)	12 weeks (*n* = 20)	
Mean age (years)	37.44 ± 9.51	49.17 ± 9.10	39.35 ± 9.64	0.021
Sex				0.458
Male, n (%)	7 (77.8)	11 (91.7)	15 (75)	
Female, n (%)	2 (22.2)	1 (8.3)	5 (25)	
History of drug abuse				0.907
Yes, n (%)	6 (66.7)	7 (58.3)	13(65)	
No, n (%)	3 (33.3)	5 (41.7)	7 (35)	
Cirrhosis status				0.041
Without cirrhosis, n (%)	7 (78)	6(50)	18(90)	
With compensated cirrhosis, n(%)	2 (22)	6 (50)	2 (10)	
Renal impairment				0.258
Yes, n (%)	0	2 (16.7)	1 (5)	
No, n (%)	9 (100)	10 (83.3)	19 (95)	
Previously treated, n (%)				0.414
Yes, n (%)	1 (11.1)	0 (0)	1 (5)	
No, n (%)	8 (88.9)	12 (100)	19 (95)	
Baseline HCV RNA (IU/mL)*	6.02 ± 1.08	5.64 ± 1.74	6.18 ± 1.12	0.840
Platelet (× 10^9^/L)	157 ± 61.23	102.2 ± 32.11	164 ± 76.57	0.321
Albumin (g/L)	43.05 ± 2.92	39.36 ± 7.22	39.62 ± 6.68	0.871
ALT (IU/L)	193.06 ± 174.35	46.82 ± 22.07	83.60 ± 82.91	0.082
AST (IU/L)	70.89 ± 47.99	102.20 ± 32.11	69.74 ± 71.22	0.647
Total bilirrubin (*μ*mol/L)	11.08 ± 6.99	11.38 ± 1.99	38.21 ± 66.51	0.585

*Abbreviations*: *ALT* alanine aminotransferase, *AST* aspartate aminotransferase, *DCV* daclatasvir, *HCV* hepatitis C virus, *SOF* sofosbuvir, *VEL* velpatasvir. * log transformations

### Virologic response

Overall, SVR 12 was achieved in 9/9 (100%; 95% confidence interval [CI], 66–100%) patients treated with SOF/VEL for 12 weeks (Fig. 2) and 31/32 (97%; 95% CI, 84–100%) with SOF/DCV for 12 or 24 weeks. The SVR12 rates of SOF/DCV 12-week, SOF/DCV + RBV 12-week, SOF/DCV 24-week, and SOF/DCV + RBV 24-week treatments were 15/15 (100%; 95% CI, 80–100%), 5/5 (100%; 95% CI, 57–100%), 10/11 (91%; 95% CI, 62–98%) and 1/1(100%; 95% CI, 21–100%), respectively (Fig. 2). RBV addition did not improve SVR (RR: 1.04; 95% CI, 0.96–1.12; *P* = 0.99). The virologic responses during and after treatment in different treatment groups were shown in Additional file 1: Table S1. No patients had virologic failure during treatment, and only one patient who was treatment-naïve, non-cirrhotic but on hemodialysis had a virologic relapse at post-treatment week 8 with SOF/DCV treatment for 24 weeks (Fig. 2).

**Fig. 3:**
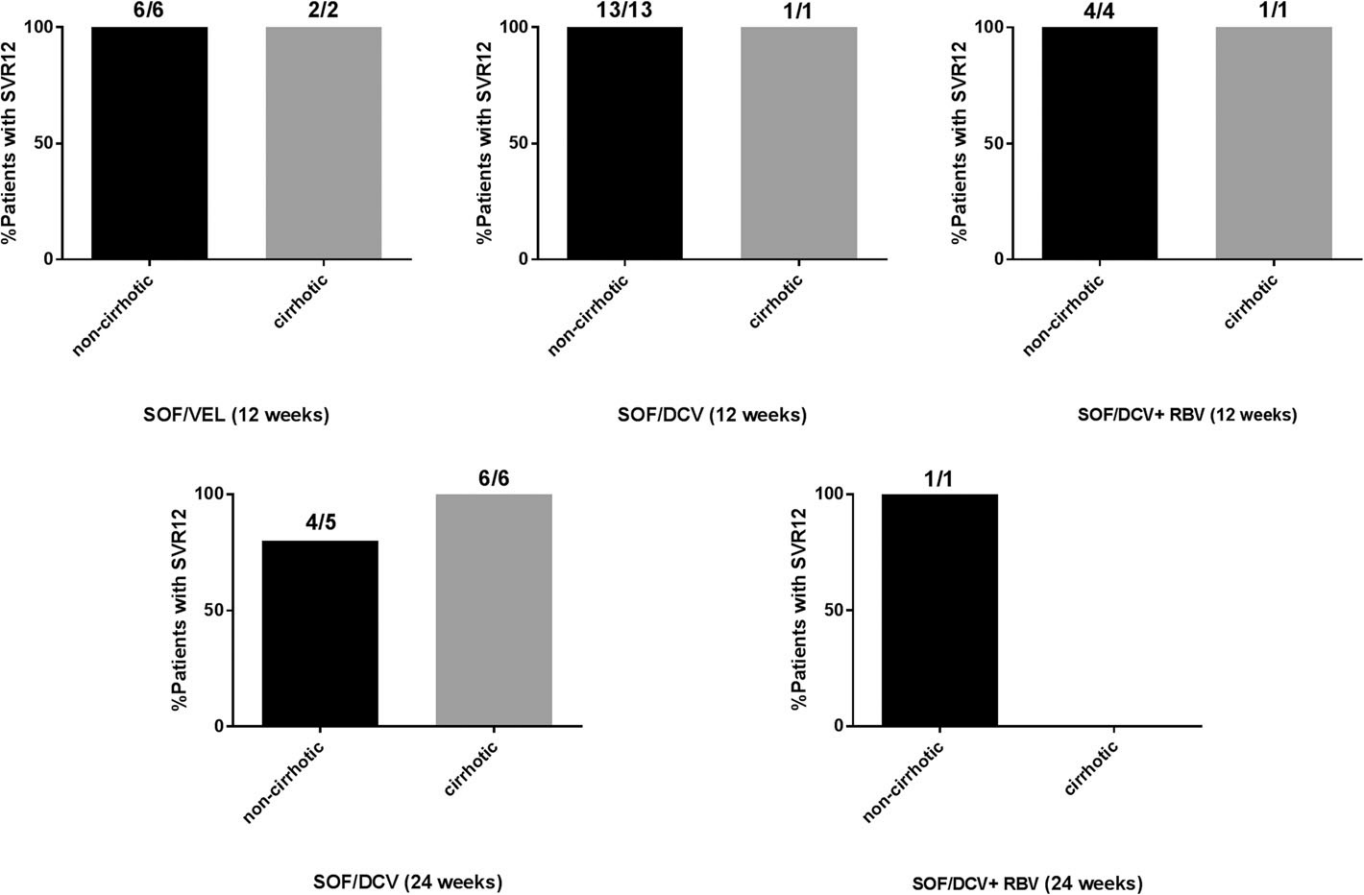
Percentage of treatment naïve patients without and with cirrhosis who have achieved SVR12 with the different treatments analyzed

Among treatment-naïve non-cirrhotic patients, the SVR12 rates of SOF/VEL 12-week, SOF/DCV 12-week, SOF/DCV + RBV 12-week, SOF/DCV 24-week and SOF/DCV+RBV 24-week treatments were 6/6 (100%; 95% CI, 54-100%), 13/13 (100%; 95% CI, 75-100%), 4/4 (100%; 95% CI, 51–100%), 4/5 (80%; 95% CI, 38–96%) and 1/1 (100%, 95% CI, 2-100%), respectively (Fig. 3). Only 1 treatment-naïve non-cirrhotic patient who was undergoing hemodialysis and treated with SOF/DCV for 24 weeks did not achieve SVR12. Among treatment-naïve cirrhotic patients, the SVR12 rates of SOF/VEL 12-week, SOF/DCV 12-week, SOF/DCV + RBV 12-week, and SOF/DCV 24-week treatments were 2/2 (100%; 95% CI, 34–100%), 1/1 (100%; 95% CI, 21–100%), 1/1 (100%; 95% CI, 21–100%), and 6/6 (100%; 95% CI, 61–100%), respectively (Fig. 3). Compared with 12 weeks of treatment, extension of SOF/DCV treatment to 24 weeks did not improve SVR (RR: 1.09; 95% CI, 0.92–1.30; *P* = 0.375).

Among the 2 treatment-experienced patients, 1 patient received SOF/VEL for 12 weeks and 1 received SOF/DCV for 12 weeks. Both of them achieved SVR12.

Among the 3 patients with renal impairment, the 2 patients on hemodialysis received the treatment of SOF/DCV for 24 weeks and the patient with chronic glomerulonephritis received SOF/VEL for 12 weeks. An end of treatment virologic response was obtained in all the 3 patients. Of the 2 patients on hemodialysis, one patient obtained SVR12, but the other patient relapsed 8 weeks after the completion of treatment with an HCV RNA level of 290 IU/mL. The patient with chronic glomerulonephritis treated with SOF/VEL for 12 weeks obtained SVR12.

The initial values of alkaline phosphatase and gamma-glutamyltransferase activities were normal at baseline and did not elevate during the treatment period and follow-up. The values of alanine aminotransferase (ALT), aspartate aminotransferase (AST) and bilirubin were slightly elevated at baseline (Table 1), decreased during treatment and normalized at the end of treatment and at 12 weeks after the completion of treatment. The values of hemoglobin, leukocytes, and platelets at treatment weeks 4, 8, and 12 or 24, and 12 weeks posttreatment did not significantly differ from baseline values.

### Adverse events

The treatment was well tolerated. Only some nonserious AEs were reported, which was probably not related to the administered drugs. The common nonserious AEs (> 5%) included headache, fatigue, insomnia, and nausea (Additional file 2: Table S2). No patients discontinued treatment prematurely because of AEs. No SAEs were recorded during the treatment period and the 12-week follow-up after treatment completion. No relevant laboratory abnormalities in bilirubin, ALT, AST, creatinine, blood urea nitrogen or estimated glomerular filtration rate (eGFR) were observed.

## Discussion

Despite revolutionary progress in the treatment of chronic HCV infection with the advent of DAAs, HCV genotype 3 infection remains a clinical challenge, especially when cirrhosis or a comorbidity is present or a previous treatment failure exists. In this study, SOF/VEL and SOF/DCV are highly efficacious and well tolerated in HCV genotype 3a-infected Chinese patients. Overall, SVR12 was achieved in 100% (9/9) of patients treated with SOF/VEL for 12 weeks and 97% (31/32) of those treated with SOF/DCV for 12 or 24 weeks with or without RBV.

SOF/VEL was recommended for treatment-naïve and treatment-experienced genotype 3 patients without cirrhosis for 12 weeks [20, 21]. The SVR 12 was achieved in 100% (9/9) patients treated with SOF/VEL for 12 weeks in this study, confirming that SOF/VEL is an efficacious therapy for genotype 3a infection. A phase 3 trial of SOF/VEL showed high overall SVR12 in genotype 3a patients [40/41 (98%)], the SVR12 in genotype 3a patients without non-structural protein 5A resistance-associated substitutions (NS5A RASs) was 100% (35/35) but the presence of NS5A RASs decreased the SVR12 to 83% (5/6) [23]. A study in Chinese patients with HCV genotype 3 infection showed that the SVR24 was 100.00% (21/21) with SOF/VEL treatment [24], but no subtype information was provided. The present study showed a similar overall SVR12 to the phase 3 trial [23]. The combination of SOF/VEL is not recommended in treatment-naïve and treatment-experienced patients infected with HCV genotype 3 with compensated (Child-Pugh A) cirrhosis because of the suboptimal results [21]. The present study only had 2 patients with cirrhosis. Both of the patients achieved SVR12. In cirrhotic and non-cirrhotic and in treatment-experienced HCV genotype 3a patients, the present study showed that SOF/VEL achieved 100% SVR12. It is suggested that SOF/VEL for 12 weeks is an efficacious and safe treatment for patients with HCV genotype 3 infection.

In this study, most of the patients (32/41) chose SOF/DCV regimen because of the availability and lower price. A phase 3 study of 12-week SOF plus DCV achieved SVR12 in 96% of patients with genotype 3 infection without cirrhosis [25]. A real-world study performed in USA showed that the SVR rate of SOF/DCV in genotype 3 was 90.8% (366/403) [26]. This study showed that prior treatment-experience and advanced liver disease were significantly predictive of reduced odds of SVR for patients with genotype 3 [26]. This study also showed that RBV did not affect cure rates of SOF/DCV treatment [26] and this is consistent with the observation in the present study. Another real-world observation carried out in Spain showed that 95% (19/20) of the HCV genotype 3 patients treated with DCV/SOF for 12 weeks achieved SVR12 [27]. This study had small number of patients compared with the study in the USA [26]. Both of these two real-world studies showed that SOF/DCV is highly effective in patients with HCV genotype 3 infection. A study in Chinese HCV genotype 3 patients showed that the SVR24 rate of SOF + DCV or SOF + DCV + RBV was 85.96% (49/57) and 91.67% (22/24), respectively [24]. The regimen demonstrated high SVR12 [97% (31/32)] for a 12- or 24- week treatment in the real-world setting of the present study. All these results therefore suggest that SOF/DCV is an efficacious therapy for HCV 3 infection.

There may be a difference between HCV genotypes 3a and 3b in the treatment efficacy of SOF-based regimens although most of the HCV genotype 3 treatment studies did not provide a differentiation between genotypes 3a and 3b. In the phase 3 trial of SOF/VEL, the overall SVR12 of SOF/VEL was 98% (40/41) in genotype 3a patients, but it was 75% (30/40) in genotype 3b patients [23]. A recent study compared the efficacy of SOF plus RBV for 24 weeks in Chinese patients with HCV genotype 3a and 3b and showed that the SVR12 rate in patients with genotype 3b [91% (62/68)] was lower than that in patients with genotype 3a [100% (58/58)] infection [28]. It is suggested that the efficacy of SOF-based regimens may differ between genotype 3a and 3b patients although the present study did not include genotype 3b patients and additional study is needed to clarify this issue.

The presence of cirrhosis may be associated with a reduced SVR12 rate in HCV genotype 3 patients compared with those without cirrhosis in the treatment of SOF/DCV [25]. In a real-world HCV genotype 3 study, DCV/SOF 12-week achieved a SVR12 of 100% in patients without cirrhosis and 90% in those with cirrhosis [27]. In a Chinese HCV genotype 3 cohort, the SVR24 rate was not statistically different between patients with and without cirrhosis (81.82% [27/33] vs. 94.20% [65/69]) [24]. In our study, 8 patients with cirrhosis used SOF/DCV treatment. SVR12 was achieved in 100% (2/2) of patients treated for 12 weeks (1 with combination of RBV) and 100% (6/6) of those treated for 24 weeks. All these results therefore suggest that SOF/DCV is also an efficacious therapy for HCV genotype 3 patients with cirrhosis although the SVR12 in patients with cirrhosis is slightly lower than in those without cirrhosis in some studies.

Prior treatment-experience may reduce the SVR for patients with genotype 3 [26]. In the phase 3 study of 12-week regimen of SOF plus DCV, the SVR12 rates were 90% (91 of 101) and 86% (44 of 51) in treatment-naïve and treatment-experienced patients, respectively [25]. In the present study, 2 patients had treatment-experience. One patient treated with 12 weeks of SOF/VEL and the other treated with 12 weeks of SOF/DCV. Both of them achieved SVR12. A cost-effectiveness analysis showed that SOF plus DCV is a safe and cost-effective option for the treatment of chronic HCV genotype 3 patients including those following a first-line treatment of peg-interferon/RBV treatment experience [29]. Therefore, SOF plus DCV is an efficacious, safe and cost-effective option for the treatment of chronic HCV genotype 3 patients with treatment-experience.

Chronic HCV infection is associated with increased chronic kidney disease (CKD) [30]. It the present study, 3 patients had renal impairment, 2 of them were on maintenance hemodialysis due to chronic renal failure and 1 had chronic glomerulonephritis and all of them were non-cirrhotic. One patient on maintenance hemodialysis and another patient with chronic glomerulonephritis achieved SVR12 with SOF/DCV 24-week or SOF/VEL 12-week treatment, respectively. One patient on hemodialysis treated with SOF/DCV for 24 weeks relapsed after treatment completion. The renal failure of this patient was related to chronic glomerulonephritis. The relationship between the chronic glomerulonephritis and HCV is unclear because of the lack of determination of HCV RNA and HCV antigen in the renal tissue. Whether the relapse is related to the alteration of immune response in renal failure of chronic glomerulonephritis is uncertain. A retrospective study evaluated the safety and efficacy of SOF/DCV in genotype 3a chronic HCV infection in 6 male patients with end-stage renal disease (ESRD) on maintenance hemodialysis (2/6 with compensated liver cirrhosis). The patients were treated with a reduced dose of SOF (one half of a 400 mg tablet) and 60 mg of DCV once daily. All of the patients completed a 12-week treatment and 100% (6/6) achieved SVR, including two with cirrhosis and two with high viral load (HCV RNA > 6,000,000 IU/mL) [31]. In our patients, the doses were not reduced considering the efficient hemodialysis in the 2 patients and the normal eGFR regardless of the glomerulonephritis in the other patient. Furthermore, studies showed that the predominant circulating inactive metabolite GS-331007 of SOF in the ESRD subjects on hemodialysis was lower when SOF was dosed 1 h before hemodialysis than when SOF was dosed 1 h after hemodialysis [32] and hemodialysis can efficiently remove the circulating metabolite GS-331007 [33]. The observation in patients on maintenance hemodialysis with HCV genotypes 1 or 2 showed that full-dose SOF (400 mg daily) have a higher SVR than reduced-dose SOF (400 mg 3 times a week) [34]. No serious adverse events or laboratory abnormalities were observed in the 3 patients with renal impairment in the present study. Therefore, although large scale study is lacking, DCV and SOF when used in a daily full-dose appear to be a safe and effective treatment in patients with HCV genotype 3 infection and renal impairment including those on hemodialysis.

RASs in HCV NS5A and non-structural protein 5B (NS5B) may affect the efficacy of DAAs. A study sequenced the NS5A and NS5B of 45 HCV genotype 3-infected patients subsequently treated with SOF ± DCV and showed that baseline NS5A RASs A30K and Y93H were detected in 9.5% of patients and the RASs in NS5A and NS5B might impact the treatment outcome [35]. A study showed that S282 T mutation in the HCV NS5B was not prevalent in DAA-treated naïve Chinese patients chronically infected with HCV including 3a and 3b [36]. A study in vitro showed that exposure to SOF may induce an escape variant with substitutions in NS5B including the RAS S282 T in genotype 3a and this escape variant may decrease the efficacy of SOF [37]. The relapsed patient in the present study was treatment-naïve and thus the possibility of presence of NS5A and NS5B RASs appears to be very unlikely although RASs detection was not performed.

Taking into account the limitations of this study including retrospective design, the small sample size of patients and the absence of pharmacokinetics profile in patients with renal impairment, prospective studies with large sample size of patients are required to further clarify the role of SOF-based regimen, especially SOF/DCV, in the treatment of chronic HCV genotype 3 infection.

## Conclusion

In this real-world study, SOF/VEL and SOF/DCV are highly efficacious and well tolerated in HCV genotype 3a-infected Chinese patients, irrespective of the presence of cirrhosis or treatment-experience. RBV coadministration and extension of SOF/DCV treatment appear to add no improvement for efficacy. These data suggest that SOF/VEL is an effective and safe treatment of HCV genotype 3a-infected patients and SOF/DCV could be an alternative treatment in the settings where preferred treatments are not available.

## Additional files

Additional file 1: **Table S1.** Virologic response (HCV RNA < 15 IU/mL) during and after treatment in different treatment groups. (PDF 43 kb)

Additional file 2: **Table S2.** Adverse events, discontinuations, and biochemical abnormalities. (PDF 44 kb)

## Data Availability

The data analyzed during the current study are available from the corresponding author on reasonable request.

## Abbreviations

AASLD: American Association for the Study of Liver Diseases
AE: Adverse events
ALT: Alanine aminotransferase
APASL: Asian-Pacific Association for the Study of the Liver
AST: Aspartate aminotransferase
CI: Confidence interval
CKD: Chronic kidney disease
DAA: Direct-acting antiviral
DCV: Daclatasvir
eGFR: estimated glomerular filtration rate
ESRD: End-stage renal disease
GLE: Glecaprevir
HCC: Hepatocellular carcinoma
HCV: Hepatitis C virus
IDSA: Infectious Diseases Society of America
LDV: Ledipasvir
NS5A: Non-structural protein 5A
NS5B: Non-structural protein 5B.
PIB: Pibrentasvir
RAS: Resistance-associated substitution
RBV: Ribavirin
RT-PCR: Reverse-transcription polymerase chain reaction
SAE: Serious adverse events
SD: Standard deviation
SOF: Sofosbuvir
SVR: Sustained virologic response
VEL: Velpatasvir
VOX: Voxilaprevir

## References

[1] Polaris Observatory HCV Collaborators (2017). Global prevalence and genotype distribution of hepatitis C virus infection in 2015: a modelling study. Lancet Gastroenterol Hepatol..

[2] Petruzziello A, Marigliano S, Loquercio G, Cozzolino A, Cacciapuoti C (2016). Global epidemiology of hepatitis C virus infection: an up-date of the distribution and circulation of hepatitis C virus genotypes. World J Gastroenterol.

[3] Xia GL, Liu CB, Cao HL, Bia SL, Zhan MY, Su CA, Nan JH, Qi XQ (1996). Prevalence of hepatitis B and C virus infections in the general Chinese population. Results from a nationwide cross-sectional seroepidemiologic study of hepatitis a, B, C, D, and E virus infections in China, 1992. Int Hepatol Commun.

[4] Bennett H, Waser N, Johnston K, Kao JH, Lim YS, Duan ZP, Lee YJ, Wei L, Chen CJ, Sievert W (2015). A review of the burden of hepatitis C virus infection in China, Japan, South Korea and Taiwan. Hepatol Int.

[5] Smith DB, Bukh J, Kuiken C, Muerhoff AS, Rice CM, Stapleton JT, Simmonds P (2014). Expanded classification of hepatitis C virus into 7 genotypes and 67 subtypes: updated criteria and genotype assignment web resource. Hepatology..

[6] Zhang Y, Chen LM, He M (2017). Hepatitis C virus in mainland China with an emphasis on genotype and subtype distribution. Virol J.

[7] Chen Y, Yu C, Yin X, Guo X, Wu S, Hou J (2017). Hepatitis C virus genotypes and subtypes circulating in mainland China. Emerg Microbes Infect.

[8] Huang K, Chen J, Xu R, Jiang X, Ma X, Jia M, Wang M, Huang J, Liao Q, Shan Z (2018). Molecular evolution of hepatitis C virus in China: a nationwide study. Virology..

[9] Jiao Y, Zhang X, Wang C, Li L, Liu J, Bar KJ, Wei H, Hu Y, Huang P, Zeng Z (2016). Hepatitis C virus subtype and evolution characteristic among drug users, men who have sex with men, and the general population in Beijing, China. Medicine (Baltimore).

[10] Ju W, Yang S, Feng S, Wang Q, Liu S, Xing H, Xie W, Zhu L, Cheng J (2015). Hepatitis C virus genotype and subtype distribution in Chinese chronic hepatitis C patients: nationwide spread of HCV genotypes 3 and 6. Virol J.

[11] Lu J, Xiang X, Cao Z, Wang W, Zhao G, Tang W, Chen L, Guo S, Zhuang Y, Shi D (2017). Younger trend of cirrhosis incidence in genotype 3 HCV infected patients in eastern China. J Med Virol.

[12] Ge D, Fellay J, Thompson AJ, Simon JS, Shianna KV, Urban TJ, Heinzen EL, Qiu P, Bertelsen AH, Muir AJ (2009). Genetic variation in IL28B predicts hepatitis C treatment-induced viral clearance. Nature..

[13] Sarin SK, Kumar CK (2012). Treatment of patients with genotype 3 chronic hepatitis C--current and future therapies. Liver Int.

[14] McCombs J, Matsuda T, Tonnu-Mihara I, Saab S, Hines P, L'italien G, Juday T, Yuan Y (2014). The risk of long-term morbidity and mortality in patients with chronic hepatitis C: results from an analysis of data from a Department of Veterans Affairs Clinical Registry. JAMA Intern Med.

[15] Shahnazarian V, Ramai D, Reddy M, Mohanty S (2018). Hepatitis C virus genotype 3: clinical features, current and emerging viral inhibitors, future challenges. Ann Gastroenterol.

[16] Probst A, Dang T, Bochud M, Egger M, Negro F, Bochud PY (2011). Role of hepatitis C virus genotype 3 in liver fibrosis progression--a systematic review and meta-analysis. J Viral Hepat.

[17] Gondeau C, Pageaux GP, Larrey D (2015). Hepatitis C virus infection: are there still specific problems with genotype 3?. World J Gastroenterol.

[18] Nkontchou G, Ziol M, Aout M, Lhabadie M, Baazia Y, Mahmoudi A, Roulot D, Ganne-Carrie N, Grando-Lemaire V, Trinchet JC (2011). HCV genotype 3 is associated with a higher hepatocellular carcinoma incidence in patients with ongoing viral C cirrhosis. J Viral Hepat.

[19] Idrees M, Rafique S, Rehman I, Akbar H, Yousaf MZ, Butt S, Awan Z, Manzoor S, Akram M, Aftab M (2009). Hepatitis C virus genotype 3a infection and hepatocellular carcinoma: Pakistan experience. World J Gastroenterol.

[20] AASLD-IDSA HCV Guidance Panel (2018). Hepatitis C Guidance 2018 update: AASLD-IDSA recommendations for testing, managing, and treating hepatitis C virus infection. Clin Infect Dis.

[21] European Association for the Study of the Liver (2018). Electronic address: easloffice@easloffice.eu; European Association for the Study of the liver. EASL recommendations on treatment of hepatitis C 2018. J Hepatol.

[22] Omata M, Kanda T, Wei L, Yu ML, Chuang WL, Ibrahim A, Lesmana CR, Sollano J, Kumar M, Jindal A (2016). APASL consensus statements and recommendation on treatment of hepatitis C. Hepatol Int.

[23] Wei L, Lim SG, Xie Q, Văn KN, Piratvisuth T, Huang Y, Wu S, Xu M, Tang H, Cheng J, et al. Sofosbuvir-velpatasvir for treatment of chronic hepatitis C virus infection in Asia: a single-arm, open-label, phase 3 trial. Lancet Gastroenterol Hepatol. 2018;(18):30343–1. 10.1016/S2468-1253. 10.1016/S2468-1253(18)30343-1 30555048

[24] Tao YC, Deng R, Wang ML, Lv DD, Yuan M, Wang YH, Chen EQ, Tang H (2018). Satisfactory virological response and fibrosis improvement of sofosbuvir-based regimens for Chinese patients with hepatitis C virus genotype 3 infection: results of a real-world cohort study. Virol J.

[25] Nelson DR, Cooper JN, Lalezari JP, Lawitz E, Pockros PJ, Gitlin N, Freilich BF, Younes ZH, Harlan W, Ghalib R (2015). All-oral 12-week treatment with daclatasvir plus sofosbuvir in patients with hepatitis C virus genotype 3 infection: ALLY-3 phase III study. Hepatology..

[26] Belperio PS, Shahoumian TA, Loomis TP, Mole LA, Backus LI (2019). Real-world effectiveness of daclatasvir plus sofosbuvir and velpatasvir/sofosbuvir in hepatitis C genotype 2 and 3. J Hepatol.

[27] Del Rio-Valencia JC, Asensi-Diez R, Madera-Pajin R, Yunquera-Romero L, Muñoz-Castillo I (2018). Interferon-free treatments in patients with hepatitis C genotype 3 infection in a tertiary hospital. Rev Esp Quimioter.

[28] Huang Rui, Rao Huiying, Xie Qing, Gao Zhiliang, Li Wu, Jiang Deyuan, Mo Hongmei, Massetto Benedetta, Stamm Luisa M., Brainard Diana M., Wei Lai (2019). Comparison of the efficacy of sofosbuvir plus ribavirin in Chinese patients with genotype 3a or 3b HCV infection. Journal of Medical Virology.

[29] Moshyk A, Martel MJ, Tahami Monfared AA, Goeree R (2016). Cost-effectiveness of daclatasvir plus sofosbuvir-based regimen for treatment of hepatitis C virus genotype 3 infection in Canada. J Med Econ.

[30] Park H, Chen C, Wang W, Henry L, Cook RL, Nelson DR (2018). Chronic hepatitis C virus (HCV) increases the risk of chronic kidney disease (CKD) while effective HCV treatment decreases the incidence of CKD. Hepatology..

[31] Sperl J, Frankova S, Kreidlova M, Merta D, Tothova M, Spicak J (2017). Combination of sofosbuvir and daclatasvir in the treatment of genotype 3 chronic hepatitis C virus infection in patients on maintenance hemodialysis. Ther Clin Risk Manag.

[32] Beinhardt S, Al Zoairy R, Ferenci P, Kozbial K, Freissmuth C, Stern R, Stättermayer AF, Stauber R, Strasser M, Zoller H (2016). DAA-based antiviral treatment of patients with chronic hepatitis C in the pre- and postkidney transplantation setting. Transpl Int.

[33] Burra P, Rodríguez-Castro KI, Marchini F, Bonfante L, Furian L, Ferrarese A, Zanetto A, Germani G, Russo FP, Senzolo M (2014). Hepatitis C virus infection in end-stage renal disease and kidney transplantation. Transpl Int.

[34] Desnoyer A, Pospai D, Lê MP, Gervais A, Heurgué-Berlot A, Laradi A, Harent S, Pinto A, Salmon D, Hillaire S (2016). Pharmacokinetics, safety and efficacy of a full dose sofosbuvir-based regimen given daily in hemodialysis patients with chronic hepatitis C. J Hepatol.

[35] Bartolini Barbara, Giombini Emanuela, Taibi Chiara, Lionetti Raffaella, Montalbano Marzia, Visco-Comandini Ubaldo, D’Offizi Gianpiero, Capobianchi Maria Rosaria, McPhee Fiona, Garbuglia Anna Rosa (2017). Characterization of Naturally Occurring NS5A and NS5B Polymorphisms in Patients Infected with HCV Genotype 3a Treated with Direct-Acting Antiviral Agents. Viruses.

[36] Qiao B, Tong YQ, Wang M, Gong WJ, Li Y (2018). Prevalence of S282T mutation in different genotypes of hepatitis C virus from DAA-treated naïve Chinese patients who were chronically infected with HCV. Eur Rev Med Pharmacol Sci.

[37] Ramirez S, Mikkelsen LS, Gottwein JM, Bukh J (2016). Robust HCV genotype 3a infectious cell culture system permits identification of escape variants with resistance to Sofosbuvir. Gastroenterology..

