# Supplementary material for: High sustained virologic response rates of sofosbuvir-based regimens in Chinese patients with HCV genotype 3a infection in a real-world setting
Source: Virol J. 2019 Jun 3;16:74. doi: 10.1186/s12985-019-1184-y (PMC6547524; doi:10.1186/s12985-019-1184-y)
Supplement: Supplementary file 1 — Table S1. Virologic response (HCV RNA < 15 IU/mL) during and after treatment in different treatment groups. (PDF 43 kb) [file 12985_2019_1184_MOESM1_ESM.pdf]

Table S1. Virologic response (HCV RNA < 15 IU/mL) during and after treatment in different treatment groups

Response	SOF/VEL, 12 weeks (n=9), n/N (%)	SOF/DCV, 12 Weeks (n=15), n/N (%)	SOF/DCV+RBV, 12weeks (n=5), n/N (%)	SOF/DCV 24 weeks (n=11), n/N (%)	SOF/DCV+RBV 24weeks (n=1), n/N (%)
During treatment					
Week 4	8/9 (89%)	13/15 (87%)	5/5(100%)	9 /11 (82%)	1/1 (100%)
Week 12	9 /9 (100%)	15/15 (100%)	5/5(100%)	10/11 (91%)	1/1 (100%)
Week 24	-	-	-	11/11 (100%)	1/1 (100%)
After treatment					
Week 12	9 /9 (100%)	15/15 (100%)	5/5 (100%)	10 /11 (91%)	1/1 (100%)
Relapse, n (%)	0	0	0	1/11 (9%)	0

Abbreviations: HCV, hepatitis C virus; IU, international units; SOF, sofosbuvir; VEL, velpatasvir; DCV,daclatasvir; RBV, ribavirin.
