# Supplementary material for: High sustained virologic response rates of sofosbuvir-based regimens in Chinese patients with HCV genotype 3a infection in a real-world setting
Source: Virol J. 2019 Jun 3;16:74. doi: 10.1186/s12985-019-1184-y (PMC6547524; doi:10.1186/s12985-019-1184-y)
Supplement: Supplementary file 2 — Table S2. Adverse events, discontinuations, and biochemical abnormalities. (PDF 44 kb) [file 12985_2019_1184_MOESM2_ESM.pdf]

Table S2. Adverse events, discontinuations, and biochemical abnormalities

Event	SOF/VEL, 12 weeks (n=9)	SOF/DCV, 12 Weeks (n=15)	SOF/DCV+RBV, 12weeks (n=5)	SOF/DCV 24 weeks (n=11)	SOF/DCV+RBV 24weeks (n=1)
Any adverse event	5 (55.6)	8 (53.3)	4 (80)	8 (72.7)	1 (100)
Discontinuation of treatment owing to adverse event, n (%)	0	0	0	0	0
Serious adverse event, n (%)	0	0	0	0	0
Common adverse event, n (%) *	5 (55.6)	8 (53.3)	4 (80)	8 (72.7)	1 (100)
Headache	1 (11.1)	1 (6.7)	1 (20)	1 (9.1)	0
Fatigue	2 (22.2)	3 (20%)	2 (40)	3 (27.2)	0
Insomnia	2 (22.2)	3 (20%)	1 (20)	3 (27.2)	1 (100)
Nausea	0	1 (6.7)	0	1 (9.1)	0
Blood bilirubin increased	0	0	0	0	0
ALT increased	0	0	0	0	0
AST increased	0	0	0	0	0
Creatinine increased	0	0	0	0	0

Abbreviations: SOF, sofosbuvir; VEL, velpatasvir; DCV, daclatasvir; RBV, ribavirin, ALT, alanine aminotransferase; AST, aspartate aminotransferase. Data are presented as n (%).*Common adverse events are those which occurred in at least 5% of patients in any treatment group.
